# An exploration of young carers’ experiences of school and their perceptions regarding their future career - a scoping review protocol

**DOI:** 10.12688/hrbopenres.13074.3

**Published:** 2020-12-07

**Authors:** Breda Moloney, Thilo Kroll, Attracta Lafferty

**Affiliations:** 1 School of Nursing, Midwifery and Health Systems, University College Dublin, Dublin, Ireland

**Keywords:** Young Carer, Perceptions, Future, School, Career, Employment

## Abstract

**Background:** Young carers are young people who care for a relative or a friend with an illness, disability, frailty, a mental health issue or addiction. Across the world, it is challenging to calculate the exact numbers due to the invisible nature of their role that can exist due to stigmatisation and fear of authoritative intrusion. As young carers reach 16 years and over, future career prospects become more significant. Young carers are more likely than their peers not to be in education, employment, or training and are more likely to do poorly at school or college than their non-caregiving peers due to the demands of caring. Recognising that positive engagement at school is a vital correlate of positive employment outcomes, young carers are at risk as their caring role can limit the range of employment opportunities open to them.

This paper outlines the protocol for a robust synthesis of the literature surrounding young carers and their career perceptions. The scoping review will address the research question ‘What is known from the literature about young carers in school and their career perceptions?’

The overall aim of this paper is to present a protocol for the scoping review to map the key concepts, types of evidence, and gaps in research related to young carers in school and their future careers.

**Methods: **The review will follow Arksey and O’Malley (2005) and Levac *et al.’s, *(2010) scoping review framework. The steps involved include: (1) research question identification; (2) relevant studies identification; (3) selection of studies; (4) data charting; (5) collating, summarising and reporting the results; and (6) stakeholders' consultation.

**Conclusions: **The scoping review is an appropriate first step to employ in presenting the literature to inform a larger research study on young carers’ experiences in school and their perceptions regarding their future careers.

## Introduction

### Recognition of young carers

The Children’s Society (2020) in the UK describe a young carer as being “a person under 18 who provides or intends to provide care for another person (of any age, except where that care is provided for payment, pursuant to a contract or as voluntary work)”. A young carer might look after someone because they're sick or have a disability or mental health issues. If their parent or family member has an alcohol or drug addiction, they may be unable to care for themselves or another person” ( The Children’s Society, 2020). Young Carers can be involved in caring that not only involves physical care, but can include psychosocial and emotional care also. Family Carers Ireland (2020) recognise a young adult carer as being in the age profile of 18–24 years and a young carer as being under the age of 18. The age of young carers in these two groups is the only difference in their overall definition. The focus of this research is primarily on the experiences of young people who are in a caring role and who attend upper level secondary education. In Ireland, the typical age range of students at this education level is between 16 and 19 years. It is therefore acknowledged those over 18 years of age in secondary school who are also in a caring role, are young adult carers and will be recognised as such throughout this research.

Recent estimates suggest there are around 7,678 young carers aged between 15 and 17 years in Ireland ( Care Alliance Ireland, 2019). It is challenging to calculate the exact numbers due to the invisible nature of their role that can exist due to stigmatisation ( Fives *et al.,* 2010) and fear of authoritative intrusion ( Rutherford *et al.,* 2013). A lack of self-identification can also result in the invisible nature of their role. Young adolescents who may see providing family care as ‘normal’, are unaware of the young carer title and do not see themselves as the one ‘in need’ ( Nap *et al.,* 2020).

In 2008, the Irish Government Office of the Minister for Children and Youth Affairs initiated a review of young carers. A research study entitled ‘ *Study of Young Carers in the Irish Population*’ was conducted by Fives *et al.* (2010), who interviewed 26 young carers from around Ireland and 30 health professionals including managers, policy staff and front-line personnel who work with young carers. The study found that there was little awareness of young carers who can ‘hide’ out of fear, and that the impact of caring can be negative when formal and informal supports are weak. The study by Fives *et al.* (2010) was the first national qualitative study of young carers, and since then, there has been no further government-directed research undertaken. With increasing recognition and lobbying on the importance of family carers, the National Carers’ Strategy (2012) was developed and recommended to “support children and young people with caring responsibilities and protect them from adverse impacts of caring” ( Department of Health, 2012, pg.14).

Improvements have been made with the formation of the ‘ *Young Carers Programme’,* set up in 2012 as part of the larger organisation ‘Family Carers Ireland’, to support carers aged 18 and under. However, there remains no national policy or strategy specifically for young carers in Ireland. Comparing with other countries, a recent European-wide Delphi study was undertaken by Nap *et al*. (2020), looking specifically at strategies to support adolescent young carers. A key recommendation of that study included the need for an integrated system involving education, social and health services being adopted by all countries. A significant theme that emerged included the need for schools’ input into identifying and supporting young carers, with some experts claiming it should be the primary responsibility of the school system to identify adolescent young carers. Limitations of school staff, funding and time within schools are challenges; however, emphasis on schools as gatekeepers and as identifiers of young carers is a significant study finding ( Nap *et al.,* 2020) and one which necessitates further exploration.

### The impact of balancing caring with education

While a proportion of research on carers highlights the benefits and skillset gained through caring such as greater maturity and compassion ( Fives *et al*., 2010), along with healthcare and advocacy skills ( Aldridge, 2014; Killam *et al.,* 2016), the majority of research on young carers in school highlight the stresses and challenges experienced when combining caring with formal education needs ( Becker & Sempik, 2019). Recent research reveals specific issues that affect young carers in school include; falling asleep in class, truanting, lack of concentration, falling behind in work ( Vizard *et al*., 2019), and poor school attendance and achievement ( Wong, 2016). The type of care delivered and the nature of the family members’ illness who the young carer supports, are proven to be significant indicators of the level of school engagement. Hamilton & Redmond’s (2020) survey with 465 young carers across primary and secondary schools in Australia highlighted that school engagement among young carers of a person with a mental illness or substance addiction is considerably lower than engagement of young carers caring for a person with a disability. This can be due to the unpredictable nature of addictive behaviours having an impact on the young carers’ routine and availability ( Hamilton & Redmond, 2020).

Across the continents, commonalities exist regarding the impact of caring on young people whilst they complete their studies. Canadian research by Stamatopoulos (2018) revealed that young carers were struggling to meet caregiving tasks alongside their educational demands. While in Australia, a case study with young carers in universities found that despite young carers pursuing their desired career choice, several reported deviating from their study and employment intentions as a result of their family caring commitments ( Day, 2019). More recent research with young adult carers in the UK by Kettell (2020) revealed that when compared with other students, young adult carers were four times more likely to give up on their education course, especially in the absence of policies and supports, where the young adult carers found it all the more challenging to complete their programs of study. Overall, these findings suggest there is a mismatch between young carers’ desire for education and career choice with the realities and challenges of being in a simultaneous caregiving role.

### Young carers and their future careers

A survey on career guidance in schools with students and guidance counsellors revealed school counsellors and parents are key influencers when deciding on career choices ( Indecon, 2019). Interestingly, the impact of those influencers varies by socio-economic group. Those from the lowest income groups are less likely to have discussed the choices with their parents compared with those from higher income groups and may therefore be in the greatest need of access to guidance counsellors. These findings can also be interrelated to the young carer experience where they commonly originate from a poorer socio-economic background ( Hill *et al.,* 2011) and where child poverty is higher amongst young carers than other children ( Vizard *et al.,* 2019). Young carers themselves may not wish to discuss their own future needs out of fear or guilt of having to leave the family home and the person they care for. In comparison with young carers’ non-caregiving peers, a school survey conducted by Code & Bernes (2006) in Canada revealed that non-young carers have a number of career concerns. These include distinct transition and career adjustment challenges related to education concerns, security, satisfaction, failing, commitment, wrong job choice, and ‘having to decide’. Those concerns can become more heightened for adolescent young carers where an added vulnerability and challenge exists in their situation of having the added responsibility of being a carer. Research conducted in the UK verifies the above findings where they assert that young carers are more likely than their peers not to be in education, employment, or training (NEET) and are more likely to do poorly at school or college than their non-caregiving peers due to the demands of caring ( The Children’s Society, 2013). It is within the school environment where young carers are amongst non-caregiving peers, and where their struggle can become more apparent. Research conducted by Choudhury & Williams (2020) confirms the importance of the relationship between young carers, school educators, and highlights the significance of schools interacting with their families in strengthening young carers’ inclusion in an educational setting.

### Rationale for research on young carers in school

Education at secondary level is becoming increasingly important around the world ( Roser & Ortiz-Ospina, 2020). Recognising that positive engagement at school is a vital correlate of positive employment outcomes ( Hamilton & Redmond, 2020), young carers are at risk as their caring role can limit the range of employment opportunities open to them. In comparison to other high income European countries, Ireland has the largest percentage of young people ( May *et al.,* 2019) with young carers predominantly in the 15–19 age category ( Central Statistics Office, 2018). Compared to younger carers, little is known about those adolescent young carers who are at a key transitional stage in their life ( Nap *et al.,* 2020).

The Irish Health Behaviour in School Aged Children Survey (2014) included a short report on the analysis of survey findings from young carers ( Callaghan *et al.*, 2016). Recommendations made called for additional support from teachers, and a person available for young carers to talk with and to seek advice from ( Callaghan *et al.*, 2016). The Children First Act (2015) outlines a number of key child protection measures that are also relevant to schoolteachers, including a statutory obligation to keep young people safe from harm. Whilst it has been recognised that young carers themselves may not self-identify as carers or choose to *hide* their caring circumstances as a result of authoritative intrusion ( Nap *et al.,* 2020) or feelings of shame ( Hamilton & Redmond, 2020), there is a level of accountability on educators to be alert to the signs of a student struggling, which could be due to the student being in a caring role. Taking young carers’ mental and physical safety into account, educators need to provide appropriate supports as much as the young carer and family are willing and able to avail of. In comparison to policies in other nations, Leu & Becker (2017) classified countries according to their level of awareness on the issue of young carers and their policy response. Ireland was classified as Level 5 Emerging - Growing Public or Specialist Awareness. It is therefore timely to continue to develop that awareness and knowledge, particularly at this time in Ireland where the impact of worldwide socioeconomic, health and demographic changes are also seen to be having an impact on young people’s education and development.

The literature outlined provides a preliminary view as to what challenges young carers face during school and how it can impact future outcomes. However, the preliminary research uncovered is diverse in its objectives and theoretical underpinnings. There is an absence of understanding when deciphering young carer perceptions of their future careers. By examining young carers’ experiences of caring and perceptions of their future careers, efforts can be made to anticipate young carers’ needs and to bridge their perceived understanding with the likely reality of balancing caring with future educational/employment demands. A scoping review protocol will outline the approach that will be adopted to determine the range of literature available that link those specific concepts. 

## Protocol

The aim of the scoping review will be to provide a collation of published and unpublished research literature on young carers in school, their experiences, and their perceptions regarding their future careers. Colquhoun *et al.* (2014) definition of a scoping review encapsulates the nature of the process that will be adhered to:

“A scoping review is a form of knowledge synthesis that addresses an exploratory research question aimed at mapping key concepts, types of evidence, and gaps in research related to a defined area or field by systematically searching, selecting, and synthesizing existing knowledge” (pg.1292–94).

For clarification and justification purposes, any variation from the protocol will be made clear and explained in the complete scoping review report as advocated by the Joanna Briggs Institute (2019). The Arksey & O’Malley (2005) and Levac *et al.* (2010) updated scoping framework will be used to conduct the scoping review. This method recognises all associated literature regardless of study design, which is incumbent for this exploration on young carers, particularly when existing carer related research tends to be fragmented ( Larkin *et al.,* 2019) and has a number of methodological challenges specific to this field of study ( Joseph *et al.*, 2019). The Arksey & O'Malley (2005) & Levac *et al.* (2010) scoping review framework is based on a literature review of services for carers of people with mental illness and therefore has credible grounding in lending itself to this topic on young carers. Steps in the framework include: (1) identify the research question; (2) identify relevant studies; (3) select studies; (4) chart the data; (5) collate, summarise and report the results; and (6) consult with relevant stakeholders. It is recognised that this process will be iterative, which will involve the researcher repeating the scoping framework steps to ensure complete coverage of the literature on young carers in school and will guide the way in which the larger research project can be designed ( Arksey & O’Malley, 2005). A key factor in the quality and desired outcome of the scoping review is based on a clear and decisive review question, which the Arksey & O’Malley (2005) framework identifies as a pivotal first step in the scoping review process.

### Scoping review research question

The research question is broad due to the emergent nature of young carer research ( Joseph *et al.,* 2019) and so as not to miss any relevant references ( Arksey & O’Malley, 2005). The research question that will guide the scoping review is thus presented:

***What is known from the literature about young carers in school and their perceptions regarding their future career?***

The “PCC” mnemonic is used as a guide to frame the scoping review question. The PCC mnemonic stands for ‘Population, Concept, and Context’ ( Joanna Briggs Institute, 2019). In this instance, young carers can be referred to as the population, the concept is related to their future careers and the context involves their experiences of family caring whilst attending school.

### Study aim and objectives

The overall aim of the scoping review is to explore the literature on young carers’ experiences of caring while attending second level education and their perceptions regarding their future career.

This will be achieved by addressing four objectives:

1. Map out the research literature, which will help to identify any gaps.
2. Examine the available evidence about young carers’ caring experiences while attending second level education and their perceptions regarding their future career.
3. Explore the extent and format of previous and current research that investigates young carers’ experiences of caring whilst attending second level education.
4. Examine the theoretical frameworks that have been used to underpin research on young carers’ experiences of caring and the multimodal factors that impact on their caring experience.

Following identification of the scoping review question and related objectives, the second component of Arksey & O’Malley’s (2005) framework involves the identification of appropriate studies to include in the review.

### Identification of relevant studies

The systematic review will be guided by the Preferred Reporting Items for Systematic Reviews and Meta-Analyses extension for Scoping Reviews (PRISMA-ScR) framework guidelines ( Tricco *et al.*, 2018). These guidelines will help the researcher and readers develop a greater understanding of the related key concepts and essential items to report on in the scoping review and will increase transparency and uptake of research findings ( Tricco *et al*., 2018). Five key electronic databases will be used to search and identify studies on young carers in the published literature.

These databases include:

- PsycINFO
- Cumulative Index of Nursing and Allied Health Literature (CINAHL) (EBSCO),
- Academic Search Complete (EBSCO)
- ERIC International Education Literature
- Applied Social Sciences Index and Abstracts (ASSIA) (ProQuest)

Relevant grey literature including family carers and young carers website information, Google Scholar and the ‘OpenGray’ literature database will be searched also. A template from the University of Toronto (2019) will be used as a guide in keeping a record of the grey literature uncovered. In relation to the exact terms to use in the search, the major concepts – *young carer, second level school and future career* will be incorporated. Synonyms from each of those concepts are included in order to capture all other literature containing related terms which other authors may have used in their literature. Boolean operators ‘AND/OR’ will be used to construct the search strategy and use of ‘Truncation*’ will also reveal related terms. Searches will combine terms as outlined in Table 1.

**Table 1.** Database search terms.

**String 1** **Young**	Young OR Adolescent OR Child OR Teenager OR Young Adult OR Juvenile OR Schoolgirl OR Schoolboy OR Youth OR Student OR Pupil
**String 2 Carer**	Carer OR Caregiver OR Care Provider OR Informal Carer OR Caring
**String 3** **Young Carer**	Young Carer
**String 4** **Second Level** **School**	Secondary School OR Secondary Education OR High School OR Senior Cycle School OR Grammar School OR Upper Secondary School OR Middle School OR School Institute OR Post-primary OR Comprehensive School OR Vocational School
**String 5** **Future Career**	Future Carer OR Career OR Future OR Prospects OR Aspirations OR Expectations OR Future Opportunities OR Livelihood OR Profession OR Workforce OR Occupation OR Job OR Life Path OR Labour OR Employ*
	**Combine String 1 & 2; results provide String 3. Combine String 3** **then with String 4 & 5** to reveal articles for title and abstract screening.

Each term will be searched for in titles and abstracts and under available subject headings e.g. Medical Subject Headings (MeSH). The *International Journal of Adolescence and Youth,* the *Journal of Career Development* and the *International Journal of Inclusive Education* will be hand searched for key words and related information on young carers and their careers, ensuring the maximum available approaches to retrieve the literature is achieved. The topics and abstracts from known young carer conference proceedings will also be hand searched to yield notable content pertaining to this research agenda. The search results from the search databases and handsearching method will be imported into *Endnote*, a bibliographic manager. Any duplicates of literature will be removed to ensure each study retrieved is not repeated, providing a more accurate final number count.

### Types of studies to select

Table 2 outlines the criteria and rationale for the inclusion and exclusion of related literature being sought for this review on young carers’ experiences and their future career perceptions.

**Table 2.** Inclusion and exclusion criteria for scoping review.

Inclusion	Rationale
Sources of evidence confined to the English language.	Searches will be limited to English language due to the costs and time involved in translation and interpretation.
Sources of evidence published between 2000 and 2020.	The search period will be confined to 2000–2020. Peer reviewed young carer research literature began to emerge from the year 2000.
Sources of evidence relating to young carers (under 18 years of age) attending upper second level education and young adult carers (18–24 years) currently attending or who have previously attended upper second level education.	The complete research study is focusing on people in upper second level education who care for a family member with a debilitating illness, disability, a mental health issue or an addiction.
Exclusion	Rationale
Sources of evidence relating to younger carers who have not completed the junior certificate cycle and primary school pupils.	The focus of the complete study is on young carers and young adult carers in secondary school who are approaching adulthood where future career prospects become more pronounced.
Carers who are attending upper level secondary school as a second attempt	The focus of research is on young carers who are in second level schooling for the first time.
Non-English language Sources of evidence	English language is the primary language spoken and read by the study reviewers. All non-English language papers will be acknowledged, and their existence documented with ‘language’ recorded as the reason for exclusion due to constraints on time, cost and translator availability ( University of York, 2009).

Original research including qualitative, quantitative, and mixed methods research, protocols, and the aforementioned grey literature material will be sourced to provide a comprehensive landscape of the available evidence ( Paez, 2017).

It is recognised there are 16–18 year old’s who provide care and who are in college or vocational training rather than school. Our study is focusing on secondary school students as indicated in our inclusion criteria however it will be acknowledged in the complete research study there is that cohort who exist outside of school and will recommend future research efforts to acknowledge their existence also.

### Data collection

The planned review will have two reviewers who will meet before the review to pre-plan as a vital requirement ( Lockwood *et al.*, 2019). The first author will be the primary reviewer of the articles, and a second reviewer will independently screen a proportion of the articles (e.g.10–20%), to ensure rigour and consistency. Relevant results will be imported into Endnote X8, a bibliographic manager, and any duplicates will be removed. Full text of relevant studies will be obtained and examined for eligibility with consideration to the inclusion, exclusion criteria and the research question ( Arksey & O’Malley, 2005). A second search using all identified keywords and index terms will be undertaken across all included databases. The final decision on articles to include will be made amongst reviewers with any conflicts settled by a third reviewer - “the tie breaker”. Numbers of studies identified, screened, assessed for eligibility, and included in the review, with reasons for exclusions at each stage, will be represented in a “Search Flowchart” as described by Moher *et al.* (2009). The primary reviewer will then be independently involved in extracting significant data, also known as charting the results. The second reviewer will chart 5–10 articles ensuring consistency of the process. A data charting form similar to the Joanna Briggs Institute (2019) guidance will be designed and adhered to by reviewers. Main headings on this charting form include study details, study design, characteristics and key findings that relate to the review question. These headings will be entered into a Microsoft Excel database, with those headed columns then populated as the relevant content is retrieved from the studies identified. A pre-trial on a sample of papers will be conducted by reviewers in adherence to a uniformed approach, as advocated by Arksey & O’Malley’s (2005) framework and as an opportunity to iron out any ambiguity. The charting will be an iterative process in which researchers will critique, agree, and update the charting form throughout as relevant ( Levac *et al*., 2010). Notes will be kept by the researcher as to the rationale for any updates to the form to ensure complete transparency in the reporting of the process undertaken and the results obtained.

### Synthesis, summary and results report

This review stage will follow the guidance set out by Levac *et al.* (2010), who advised a review must involve a descriptive numerical summary and a thematic analysis. In addition, they recommended three distinct steps to increase consistency, which this research will adhere to:

- Reporting results
- Analysing the data
- Applying meaning to the results.

Following Arksey & O’Malley’s (2005) method, the results will be presented by descriptive numerical analysis through the use of displaying tables and charts, featuring spread of studies by year, origin, area of focus (clinical, policy, educational, health impact etc.), research methods and key findings. This will include a narrative thematic analysis summary explaining in what way the results relate to the scoping review question and objectives, which sought information on the scope of literature on young carers in second level education and their future careers. Data analysis will occur through descriptive thematic analysis, discussion, and critique with the researchers along with cross referencing with the ‘charting form’ content as described in stage four of this protocol. As advised by Nyanchoka *et al*. (2019), an evidence gap map to improve research planning and strategic research prioritization will also be provided. Given the aim of this scoping study is on comprehensive coverage, rather than the quality of the evidence, the researcher deduces this stage will deliver on its intended goal to map the literature and identify gaps in the literature that future research can address. The final component in Arksey & O’Malley’s (2005) framework involves reflection and discussion with relevant stakeholders.

### Consultation with relevant stakeholders

Initial findings from the scoping review will be presented to a number of key stakeholders. These include young carers themselves and a number of other personnel including social workers, pastoral care, and school guidance counsellors, who are in a position of support and advocacy for young people and who may have encountered individuals who are in a caring role. This component will be to provide an opportunity for stakeholders to give first-hand feedback beyond that found in the literature, and to allow the researcher subjective engagement with participants reinforcing integrities and motivating factors for the larger research study. Young carers will be accessed by way of carer consultation groups - Family Carers Ireland, Youth Service Ireland, Pastoral Care Youth and Foroige Youth group. A community based social worker group, pastoral care group and a number of school guidance counsellors in the locality will be approached by the primary author. Contact will be made by the primary author phoning the designated manager of these groups with an introduction and explanation of the research topic. Permission will be sought for an informal meeting to share the complete scoping review findings and to gain feedback from those group participants. After the initial phone contact, a follow up email will also be sent to each of the managers outlining the request in detail and to confirm their availability to attend a meeting at their workplace, where further explanation and results of the scoping review will be shared.

### Dissemination

Findings will be disseminated through the primary author contacting the aforementioned carer group managers, completion of a peer reviewed publication, within the researchers’ university research affiliations, and through the international young carer conference which will be held in Brussels in 2021. Strict adherence to ethical procedures in the dissemination of information particularly when involving young people will be adopted by the researchers. In addition, public health policy and procedures will be adhered to in dissemination of the findings with guidance on any ethical queries with full ethical approval being sought from the authors affiliated University Ethics Committee.

### Study status

The study is at Stage 2 - Identification of relevant studies. A review of reference management software has begun with a preliminary incorporation of search terms into the aforementioned search engine databases.

## Discussion

The scoping review protocol is an essential component to adopt to structure and organise a scoping literature review. This is particularly relevant when the information on a topic is complex and diverse ( Lockwood *et al.*, 2019; Peters *et al.,* 2015; Sucharew & Macaluso, 2019), such as in this research on young carers where it is envisaged the literature will be diverse across many disciplinary fields including health, education, and social policy. This scoping review protocol provides a robust method for exploring related research studies, amalgamating evidence, and identifying gaps in the literature. Each of Arksey & O’Malley’s (2005) measures shall be applied with regard to the process of conducting the scoping review on young carers’ experiences and their perceptions regarding their future careers. Clear, specific recommendations for future research based on gaps in knowledge identified from the results of the review will be presented in the scoping review report.

## Data availability

No data are associated with this article.
