# Supplementary material for: An exploration of young carers’ experiences of school and their perceptions regarding their future career - a scoping review protocol
Source: HRB Open Res. 2020 Dec 7;3:41. Originally published 2020 Jun 25. [Version 3] doi: 10.12688/hrbopenres.13074.3 (PMC7527861; doi:10.12688/hrbopenres.13074.3)
Supplement: Peer review history

# Peer review: Reviewer response for version 2

**DOI:** 10.21956/hrbopenres.14311.r27962

**Published:** 2020-11-23

**Authors:** Kate Blake-Holmes

**Affiliations:** 1 School of Social Work, University of East Anglia, Norwich, UK

This is a very important topic and this scoping review provides a excellent summary of the research and the evidence of its significant impact on the lives of young carers and young adult carers.  

 The paper gives a good summary of the recognition and support available to young carers in Ireland.  

 It would be interesting to explore if any papers reviewed discussed an intersection of vulnerability for young carers from marginalised groups such as BAME communities, refugees and traveller communities.

Is the study design appropriate for the research question?

Yes

Is the rationale for, and objectives of, the study clearly described?

Yes

Are sufficient details of the methods provided to allow replication by others?

Yes

Are the datasets clearly presented in a useable and accessible format?

Not applicable

Reviewer Expertise:

Parental Mental Illness, young carers

I confirm that I have read this submission and believe that I have an appropriate level of expertise to confirm that it is of an acceptable scientific standard.

# Response

**Published:** 2020-11-26

**Authors:** breda moloney

**Affiliations:** University College Dublin, Ireland

The authors would sincerely like to thank you Dr Blake-Holmes for your feedback on this scoping review protocol. Those young carers from any marginalised groups e.g. BAME, refugee or traveller communities is certainly a vital component and one which the authors will highlight should this arise from the literature uncovered. The methodology incorporated for the larger research study will be aligned accordingly to account for inclusion and recognition of young carers from any marginalised groups.

# Peer review: Reviewer response for version 1

**DOI:** 10.21956/hrbopenres.14173.r27595

**Published:** 2020-09-30

**Authors:** Polly Vizard, Kerris Cooper

**Affiliations:** 1 Centre for Analysis of Social Exclusion, London School of Economics and Political Science, London, UK; 2 Centre for Analysis of Social Exclusion, London School of Economics, London, UK

This is an important area of research. The scoping review will provide a systematic examination of the available evidence on the experiences of young carers in school and their perceptions regarding their future career. This will be informative in its own right and will provide a foundation for further research. In addition, as the authors note, there is increasing recognition that teachers and schools can play a key role in identifying and supporting young carers, and the review is potentially important in informing the ongoing discussions and policy developments on this issue.

 Our overall assessment is that the methods are appropriate and well specified. The study protocol outlines a robust approach to a scoping review which will ultimately identify and bring together evidence filling an important gap in the literature on young carers and informing policies aimed at addressing educational and employment disadvantages faced by young carers.

 We have several minor suggestions relating to definitions, scope and methods, which we set out below.

 First, we are unclear whether the definition of young carer put forward at the beginning of the abstract is the working definition of young carer being proposed in the project as a whole (and in particular as a basis for search terms being adopted for the scoping review). This definition defines young carers as individuals under 18 years old. While this definition reflects the statutory definitions and definitions adopted for many policy purposes (e.g. in relation to identification and assessment of children’s support needs within the context of local authority children’s services), it excludes those aged 18 or above. However, for this research, it would seem highly relevant to be examining and building up evidence on the participation of young carers who are 18 year olds in compulsory education (the school leaving age is now 18 in England for example) and this seems to be implied by the other sections of the proposal. Including evidence on participation and transitions into 18+ education, training and work careers might also be important. A broader definition of young carers, to include young carers who are 18 years old, and potentially young adult carers aged 18-21, seems to fit with the discussion in the full proposal.

 In addition, the definition of young carers set out in the abstract refers to individuals who are undertaking caring activities for relatives. In some instances however it is possible that young carers are providing care for individuals who are not relatives - so again a broader definition would be relevant.

 Second, in terms of the circumstances and experiences of young carers in school, it would be interesting to identify and review any available specific evidence on exclusions, absenteeism and lateness, since all three can be indications that children are struggling at school. Data of all three is available in school records.

 While the proposal focuses on ‘school’, 16-18 year olds might be in college or work based training rather than school. It will be important to specify search terms in a way that ensures that any available evidence relating to young carers or young adult carers in the college context is included within the review. Any research gaps relating to the perspective of 16-18 year olds who provide care and who are in college or vocational training rather than school should be identified as part of the scoping review.

 Third, in terms of the methods used for the scoping review, one important question that would benefit from a more detailed response is what counts as ‘evidence’ and relatedly how the quality of the evidence will be evaluated and different types of evidence synthesised. Casting a wide net to ensure all evidence is included on this topic which is so far relatively under-explored is appropriate, but when including such a mix of different types of evidence including websites and conference proceedings, some thought needs to be given as to how findings from these different types of evidence will be integrated when mapping the literature and ‘applying meaning to the results’. It is important to evaluate the evidence that is identified in order to answer the research question ‘What is known from the literature about young carers in school and their perceptions regarding their future career?’

 A number of steps can be taken in relation to this. Firstly, the inclusion criteria may be iteratively developed as material is found in order to more clearly distinguish for example what kind of website material will be included. Secondly, study design should be included as a main heading in the charting form so that evidence can be easily grouped depending on the level of robustness. Thirdly, in analysing the data and applying meaning to the results the different types and quality of evidence should be acknowledged, with more emphasis placed on findings from more robust studies. An example of this approach can be found in the section ‘a hierarchy of methodologies’ in Cooper K and Stewart K (forthcoming) ‘Does household income affect children’s outcomes? A systematic review of the evidence’, *Child Indicators Research*.

 Fourth, a few minor comments related to conducting the searches. The protocol outlines a number of strategies to ensure as wide a search as possible to identify relevant material. In addition to those already mentioned I would recommend the use of snowballing all relevant references found in the studies reviews, as in Greenhalgh, T., & Peacock, R. (2005) ^1^. The authors found this method identified many more relevant studies than from searches alone, and this is unsurprising given some of the biases of particular databases.

 A couple of additional search terms that may be relevant to add to String 5 are: aspiration and prospects.

 Finally, we note that there is no research ethics statement included in the proposal, but that the researchers are proposing meeting with children. It is of course essential that the standard relevant ethics procedures and any child protection clearances that are necessary are complied with.

Is the study design appropriate for the research question?

Yes

Is the rationale for, and objectives of, the study clearly described?

Yes

Are sufficient details of the methods provided to allow replication by others?

Yes

Are the datasets clearly presented in a useable and accessible format?

Not applicable

Reviewer Expertise:

NA

We confirm that we have read this submission and believe that we have an appropriate level of expertise to confirm that it is of an acceptable scientific standard.

## References

[1] : Effectiveness and efficiency of search methods in systematic reviews of complex evidence: audit of primary sources. *BMJ*.2005;331(7524) : 10.1136/bmj.38636.593461.68 1064-5 10.1136/bmj.38636.593461.68 16230312PMC1283190

# Response

**Published:** 2020-11-02

**Authors:** breda moloney

**Affiliations:** University College Dublin, Ireland

The authors would like to thank you for your comments to our scoping review protocol and for your suggestions. We have responded to your comments below:

- A broader definition of young carers will be provided which will be more aligned to the young carer profile as discussed throughout the protocol.
- It is envisioned the scoping review will highlight the major emergent themes arising out of the literature on young carers in school. Where a preliminary review has highlighted absenteeism and lateness issues for young carers, we will consider this significance in the methodological approaches following the complete scoping review.
- We acknowledge there are 16-18 year old’s who provide care and who are in college or vocational training rather than school. Our study is focusing on secondary school students as indicated in our inclusion criteria however it will be acknowledged there is that cohort who exist outside of school and will recommend future research efforts to acknowledge their existence also.
- In relation to reviewing the quality of the evidence literature uncovered, the different types of literature will be made known in the scoping report through the adherence to the ‘Joanna Briggs Institute’ guidance on evidence transparency using the PRISMA-ScR Checklist (Preferred Reporting  Items for Systematic reviews and Meta-Analyses Extension for Scoping Reviews). We will be transparent and explicit in the approach we will take, including justifying our approach and clearly documenting analyses. The inclusion criteria will be iteratively developed as evidence is uncovered in order to clearly distinguish the type of material included e.g. journals or websites. The study design will be included as a main heading in the charting form to demonstrate and categorize the different types of evidence along with highlighting more robust studies.
- Additional search terms ‘aspiration’ and ‘prospects‘ will  be added to String 5
- Within the research context, full ethical approval will be sought from the authors affiliated University along with strict adherence to national and local public health policy and procedures.

# Peer review: Reviewer response for version 1

**DOI:** 10.21956/hrbopenres.14173.r27656

**Published:** 2020-08-18

**Authors:** Gerry Redmond

**Affiliations:** 1 College of Business, Government & Law, Flinders University, Adelaide, Australia; 2 Social Policy Research Centre, University of New South Wales, Sydney, Australia

Literature on young carers is summarised very well. However, the first part of the research question 'What is known from the literature about young carers in school.." seems a bit broad to me. For example, will the study mainly focus on academic engagement, or will it also focus on engagement with peers, bullying and other factors? The study could perhaps start with an examination of different forms of engagement (e.g.., see the well cited paper Fredricks *et al.* (2004) ^1^). Alternatively, the study could focus on peer relations, or family support, and how these feed into aspirations.

 Search databases should include Google Scholar, as it will capture a lot of the gray literature.

 Search method is comprehensive and rigorous.

 It's really positive to see that findings will be reported to key stakeholders, including young carers. It will be important to hear their views on what they see as important issues for their school life and future aspirations. 

 Note that the definition of young carers seems to be limited to those providing physical care. An important part of caring is provision of emotional care.

Is the study design appropriate for the research question?

Yes

Is the rationale for, and objectives of, the study clearly described?

Yes

Are sufficient details of the methods provided to allow replication by others?

Yes

Are the datasets clearly presented in a useable and accessible format?

Not applicable

Reviewer Expertise:

Young people's wellbeing in the context of marginalisation.

I confirm that I have read this submission and believe that I have an appropriate level of expertise to confirm that it is of an acceptable scientific standard.

## References

[1] : School Engagement: Potential of the Concept, State of the Evidence. *Review of Educational Research*.2004;74(1) : 10.3102/00346543074001059 59-109 10.3102/00346543074001059

# Response

**Published:** 2020-11-02

**Authors:** breda moloney

**Affiliations:** University College Dublin, Ireland

The authors would like to thank you for taking the time to read our scoping review protocol, for your positive feedback and recommendations.

- The nature of scoping reviews is to capture a large body of literature pertaining to a broad topic and therefore the research question adopts this approach also.
- Google scholar will also be included in the grey literature search.
- A broader definition of young carers to include the psychosocial and emotional elements of caring will be provided which will be more aligned to the young carer profile as demonstrated throughout the protocol.
